# Emotional Disturbances and Crohn's Disease

**DOI:** 10.2174/0117450179410096250926135151

**Published:** 2025-10-02

**Authors:** Mara Lastretti, Ettore D'Aleo, Roberta Pica, Daniela De Nitto, Mauro Giovanni Carta, Gavino Faa

**Affiliations:** 1 Department of Economic, Psychological and Communication Sciences, Niccolò Cusano University, Rome, Italy; 2 IBD Center - Gastroenterology Unit at Sandro Pertini Hospital, Rome, Italy; 3 Department of Medical Sciences and Public Health, University of Cagliari, Cagliari, Italy; 4 Department of Biology, College of Science and Technology, Temple University, Philadelphia, PA, USA

**Keywords:** Crohn’s disease, Psychological assessment, Gut-brain axis, Cognitive impairment, Anxiety, Depression, Inflammatory bowel disease, Biopsychosocial model

## Abstract

**Introduction:**

This review aims to explore the psychological, psychiatric, and neurocognitive dimensions of Crohn’s disease. We examine the bidirectional interactions between the gut and brain, analyze the most widely used psychological assessment tools, and discuss current intervention models.

**Methods:**

A comprehensive narrative review was conducted, integrating the literature research findings of , psychosomatic medicine, neuropsychology, psychoneuroimmunology, and gut-brain axis. Attention was given to validated psychometric tools and emerging therapeutic approaches.

**Results:**

The evidence reveals a consistent link between CD and emotional disturbances, cognitive deficits, and altered gut-brain communication. Common neuropsychological impairments include deficits in attention, memory, and executive functioning. Anxiety, depression, and alexithymia are prevalent in CD patients and are associated with worse disease outcomes. Cognitive Behavioral Therapy (CBT), Acceptance and Commitment Therapy (ACT), and mindfulness-based interventions have demonstrated efficacy in enhancing both psychological well-being and disease management.

**Discussion:**

Findings confirm the link between Crohn’s disease, emotional disturbances, and cognitive deficits, highlighting the need to distinguish between primary impairments of inflammatory/neurobiological origin and those secondary to psychological distress or low illness insight. Key gaps remain regarding how these mechanisms interact over time and the long-term effects of psychological interventions. Integrating psychological assessment and support into clinical care is crucial for enhancing adherence, resilience, and overall quality of life.

**Conclusion:**

Understanding CD through a biopsychosocial lens highlights the necessity of integrating psychological assessment and intervention into standard IBD care. Early identification and tailored treatment of emotional and cognitive disturbances can significantly improve patients’ quality of life and overall clinical outcomes.

## 1 INTRODUCTION

Patients affected by Crohn’s disease (CD), a chronic condition primarily affecting the gastrointestinal tract, show a high incidence of emotional disturbances, including psychosocial, behavioral, and psychiatric symptoms [1]. A bidirectional relationship is observed between emotional disturbances and gastrointestinal symptoms. Interestingly, emotional disturbances detected in CD patients are similar to those observed in patients with ulcerative colitis, another group of diseases classified as inflammatory bowel disease (IBD) [2].

The Cornell Medical Index has been identified as a useful tool for screening patients affected by IBD, aiming to enable early identification of emotional disturbances in those with CD. Pioneering studies investigating the psychological status of CD patients—using tools such as the Manifest Anxiety Scale and the Eysenck Personality Inventory—showed that CD patients were significantly more neurotic and more anxious compared to control subjects, suggesting that CD could be classified as a psychosomatic illness [3].

Recently, a four-year follow-up study assessing the psychosomatic profile of 111 IBD patients revealed that more than half maintained psychosomatic syndromes during the follow-up period. In particular, demoralization, allostatic overload, and type A behavior represented persistent psychological burdens typical of IBD patients, including those with CD, highlighting the need for psychosomatic evaluation in this population [4].

This article aims to review psychological, psychiatric, and neurocognitive comorbidities in patients with CD, and to evaluate the psychological impact of CD in terms of depression and anxiety through the use of questionnaires and clinical interviews. We also analyzed the most commonly used psychological intervention models, with a focus on their methodologies and clinical applications [5].

## 2 METHODOLOGY OF THE NARRATIVE REVIEW

This narrative review was conducted to synthesize current evidence on the psychological and neurocognitive dimensions of Crohn’s disease, with a specific focus on mood disorders, cognitive impairment, insight, and psycho-therapeutic approaches. A non-systematic literature search was performed using the databases PubMed, Scopus, and PsycINFO, covering publications from January 2000 to March 2025. Keywords included combinations of “Crohn’s disease”, “inflammatory bowel disease”, “psychology”, “cognition”, “depression”, “anxiety”, “alexithymia”, “insight”, “gut-brain axis”, and “psychotherapy”. Studies were selected based on relevance to the topic, availability in English or Italian, and inclusion of clinical populations. Exclusion criteria included animal studies, purely mechanistic biomedical research without psychological correlates, and duplicate publications. The SANRA (Scale for the Assessment of Narrative Review Articles) framework was used as a guideline to enhance transparency and methodological quality. Although the narrative design does not permit meta-analytic conclusions, it allows a broad and integrative synthesis of heterogeneous findings across disciplines [5].

### 2.1 Background and Clinical Overview

Crohn’s disease (CD) is a lifelong inflammatory condition of unknown etiology, likely resulting from complex interactions between genetic, environmental, and immunological factors. It causes chronic, segmental inflammation of the gastrointestinal tract and can affect any region from the mouth to the anus, although it is most prevalent in the terminal ileum and proximal colon. CD belongs to the group of inflammatory bowel diseases (IBD), which includes ulcerative colitis (UC), indeterminate colitis (IC), and inflammatory bowel disease unclassified (IBDU).

Unlike UC, which presents with continuous lesions confined to the rectum and colon, CD is characterized by skip lesions—segments of inflamed tissue interspersed with healthy areas. This hallmark feature often complicates both diagnosis and disease management, particularly due to its transmural nature and systemic manifestations.

Diagnosis of CD relies on a combination of clinical evaluation, biochemical markers, endoscopic visualization, histological findings, and radiological imaging. Common symptoms include abdominal pain, diarrhea, weight loss, fatigue, and rectal bleeding. Fistula formation—connecting disparate parts of the gastrointestinal tract or extending externally—is a known complication that reflects the disease’s transmural involvement.

Histologically, early mucosal lesions often include aphthous ulcers, micro-ulcerations, and epithelial necrosis, usually centered on lymphoid follicles. One of the most specific histological features is the presence of non-caseating epithelioid granulomas, although these are not exclusive to CD and may be observed in other infections such as tuberculosis, Yersinia, or Campylobacter colitis. For this reason, differential diagnosis must include Ziehl–Neelsen staining, particularly in tuberculosis-endemic areas [5].

Research on CD pathogenesis points to several over-lapping mechanisms: genetic susceptibility [6, 7], dys-regulation of the intestinal immune system [5], barrier dysfunction, microbiota imbalances (dysbiosis) [8, 9, 10], and environmental exposures such as cesarean delivery or early antibiotic use [11, 12]. High-fat, low-fiber diets have also been implicated [13].

While these findings offer plausible mechanistic explanations, many studies stop at descriptive correlations without critically evaluating how these factors interact to influence the disease’s progression or therapeutic response. For instance, the hypothesized role of *Mycobacterium avium* subspecies *paratuberculosis* (MAP) in CD remains controversial and lacks conclusive evidence [14].

Furthermore, although epithelioid granulomas are useful in differentiating CD from UC (where they are typically absent), their prognostic relevance remains unclear. Other histological markers, such as basal plasma-cytosis and transmural fibrosis, underscore the immune-mediated nature of the disease [15].

Recent findings increasingly suggest that CD’s impact extends beyond the gastrointestinal system, involving neuropsychological domains. Patients often report symptoms such as cognitive slowing, memory issues, and decreased executive functioning—phenomena likely influen-ced by both inflammation-driven neurodegeneration and comorbid mood disorders.

In this context, it is crucial to differentiate primary cognitive deficits, potentially resulting from systemic inflammation or immune dysregulation, from secondary deficits, which may be linked to low insight into illness, poor treatment adherence, and depression. These secondary mechanisms can, in turn, perpetuate disease activity, forming a vicious cycle of poor control of the disease and worsening of cognitive function.

Depression and anxiety, prevalent among CD patients, not only reduces the quality of life but may also impair insight and executive functioning, thus compromising adherence to treatment regimens (*e.g*., diet, medication compliance). Consequently, neurocognitive impairment and poor insight are often observed even in clinically “stabilized” patients, suggesting that preserved neurocognition may be a prerequisite for accurate disease perception and behavioral adaptation.

Thus, quantification alone is insufficient. A more nuanced, qualitative assessment of cognitive and emotional complaints—interpreted through the lens of disease stage—is essential for developing tailored therapeutic strategies. Such an approach may include neurocognitive rehabilitation, psychoeducation, and interventions aimed at restoring insight and emotional resilience.

### 2.2 The Gut-Brain axis in Crohn’s Disease

The gut–brain axis represents a complex bidirectional communication system involving hormonal, microbial, metabolic, immunological, and neural signals, linking intestinal and cerebral functions in a dynamic interplay. Emerging evidence indicates that cellular and molecular signaling from the gut can modulate brain activity, thereby establishing a crucial connection between gastrointestinal and neurological disease status. Notably, microbial metabolites have been found to modulate the interaction between microglia and astrocytes, clearly demonstrating the influence of the intestinal microbiota on central nervous system (CNS) cell function [16].

Inflammatory bowel diseases (IBD), including Crohn’s disease (CD), have been increasingly associated with neurodegenerative processes. Multiple studies have reported alterations in the gut microbiome linked to the onset and progression of neurodegenerative diseases such as Parkinson’s disease (PD) [17, 18]. Specifically, CD and other IBDs have been shown to increase the risk of developing PD, a finding replicated across different populations [19, 20, 21]. Furthermore, a recent meta-analysis confirmed a moderately increased risk of both dementia and PD in patients with CD [22].

The relationship between CD and the CNS extends beyond neurodegeneration. CD patients are at increased risk for demyelinating diseases, including multiple sclerosis, highlighting the possibility of shared inflammatory or immune-mediated mechanisms [23, 24]. Additionally, the strong functional and anatomical connection between the brain and gut is supported by clinical evidence that intestinal malabsorption syndromes, particularly those involving vitamin B12 deficiency, can lead to neurologic and neuropsychiatric symptoms such as cognitive decline and psychosis [25].

At a molecular level, cytokines released by the intestinal mucosa in response to inflammation in CD can enter systemic circulation, cross the blood–brain barrier, and affect neuronal and glial cell function. Similarly, microbial metabolites and enteric nervous system signaling can influence distant organs such as the brain, potentially promoting neuroinflammation and contributing to neurodegeneration [26].

Importantly, this body of evidence calls for a holistic approach to CD, recognizing that emotional disturbances, depression, anxiety, and impaired insight into illness are not merely psychiatric comorbidities, but integral components of gut–brain axis dysfunction. These symptoms may reflect underlying neurocognitive deficits that are either primary (due to immune or microbial factors) or secondary to low treatment adherence and chronic disease progression. Thus, beyond quantifying cognitive symptoms, it becomes essential to qualify and interpret them within the broader context of disease stage, mood state, and insight, to develop more personalized and effective therapeutic strategies.

### 2.3 The Link Between Crohn’s Disease, Gut Microbiome, and Mental Health

In patients with Crohn’s disease (CD), mental health symptoms such as anxiety and depression may arise from a multifactorial etiology. On one side, these symptoms can be triggered by the psychological burden of coping with a chronic, unpredictable disease of unclear etiology and pathogenesis, characterized by abdominal cramping, pain, diarrhea, weight loss, restrictive diets, and chronic fatigue [27]. This stress can significantly affect emotional well-being and reduce quality of life, especially when compounded by low insight into the illness, which may in turn impair treatment adherence and long-term disease management.

On the other hand, increasing evidence supports the involvement of biological mechanisms, particularly those involving the gut–brain axis, in the emergence of neuropsychiatric symptoms in CD [28]. The bidirectional communication between the gastrointestinal tract and the central nervous system is crucial for maintaining neuropsychological balance. In healthy conditions, the intestinal microbiota contributes to the synthesis of neurotransmitters and hormones such as serotonin and gamma-aminobutyric acid (GABA), which are fundamental for emotional regulation, mood stability, and cognitive processes [29]. Furthermore, the gut microbiota plays an essential role in preserving the integrity of the intestinal barrier, thereby preventing the translocation of pro-inflammatory substances into systemic circulation and the central nervous system [30].

However, in CD patients, this balance is often disrupted. Dysbiosis—an imbalance in the composition and function of the intestinal microbiota—is a frequent finding in IBD and is particularly notable in CD. This condition is associated with reduced microbial diversity, increased intestinal permeability (“leaky gut”), and heightened systemic inflammation. A specific hallmark of dysbiosis in CD is the increased abundance of adherent-invasive *Escherichia coli* (AIEC), which aggravates inflammation and disrupts mucosal integrity through the production of pathogenic metabolites [31].

This cascade of events can lead to neuroinflammation, which has been implicated in structural and functional changes in the brain and is increasingly recognized as a contributor to depression and other cognitive deficits [32]. These impairments may, in turn, reduce insight into the disease, further hindering treatment adherence and perpetuating a cycle of poor disease control and psycho-logical dysfunction. Notably, recent longitudinal studies suggest that increased intestinal permeability may precede the clinical onset of CD by several years, supporting the hypothesis that gut barrier dysfunction is not only a consequence but also a potential trigger for the development of the disease [33].

In this context, understanding whether mental health symptoms in CD are primary (linked to gut-brain interactions and inflammation) or secondary (resulting from low insight and coping difficulties) is critical. A comprehensive approach should integrate psychological, neurocognitive, and biological domains, aiming not only to quantify symptoms but to interpret them within a phase-specific model of disease progression and patient awareness.

### 2.4 The Gut-Brain Axis Paradigm

The principal components of the gut–brain axis are the enteric nervous system (ENS), the vagus nerve, and the gut microbiome. Vago–vagal reflexes interconnect the two “brains”—the central nervous system (CNS) and the ENS—through complex networks of motor and sensory fibers, enabling continuous bidirectional communication between the gut and the brain [34]. This neuroanatomical and functional link is particularly relevant in chronic diseases such as celiac disease (CD), where both mood regulation and cognitive function are often compromised.

Motor fibers of the vagus nerve convey central commands that regulate gastrointestinal processes, including intestinal motility, gastric secretion, and mucosal barrier integrity. In parallel, sensory afferents transmit information from the gut to the brain regarding nutrient content, satiety, inflammation, and visceral discomfort [35 ]. Under physiological conditions, this dynamic feedback loop coordinates vital processes such as peristalsis, immune modulation, and nutrient absorption. However, when this axis is disrupted—as observed in CD—dysfunctions in both the ENS and vagal signaling may emerge, with downstream consequences on neurocognitive function and patient insight into the disease.

Notably, the ENS is increasingly recognized as a therapeutic target in CD patients due to its regulatory role over both gut physiology and brain-related outcomes [36]. Impairments in this system can influence neuro-psychological health, potentially contributing to cognitive deficits, depressive symptoms, and reduced insight—all of which are known to affect treatment adherence. Despite these associations, the precise molecular pathways underlying ENS and vagal dysfunction in CD remain incompletely understood [37, 38], warranting a more critical exploration of existing models. Understanding whether these neuroenteric changes precede or result from poor adherence, mood alterations, or inflammation could refine our approach to symptom interpretation, particularly across different stages of the disease.

### 2.5 Neuropsychological And Psychological Disorders In Crohn’s Disease: An Overview

Recent scientific literature has increasingly high-lighted the intricate relationship between the development and function of the nervous system and the composition of the gut microbiome. Importantly, this interaction is not unidirectional: the central nervous system (CNS) also plays an essential regulatory role over the gut microbiota, forming a dynamic, bidirectional communication system commonly referred to as the gut–brain axis. Multiple interrelated pathways mediate this crosstalk, including immune signaling, neuroendocrine mechanisms, and vagus nerve activation.

There is a growing body of evidence linking gut microbiota dysregulation (dysbiosis) to a broad spectrum of neuropsychiatric and neurodevelopmental disorders, such as Alzheimer’s disease, Parkinson’s disease, and autism spectrum disorder, as well as mood-related conditions like anxiety and depression [39]. This suggests that changes in microbial diversity and composition can influence brain function and behavior through systemic and localized inflammatory responses, altered neuro-chemical production, and disrupted barrier functions.

In this context, investigating gut–brain axis dys-regulation in the setting of chronic inflammatory bowel diseases (IBD)—particularly Crohn’s disease (CD)—is especially relevant. Patients with CD often exhibit neurocognitive impairments and psychological symptoms; however, the exact pathophysiological mechanisms remain poorly elucidated. A more critical understanding is required to distinguish between primary cognitive deficits, potentially driven by systemic inflammation and microbial imbalance, and secondary deficits that may arise due to mood disorders or impaired insight into the disease.

Notably, IBD has been consistently associated with a decrease in gut microbial diversity and an increase in pro-inflammatory species such as *Escherichia coli* and other members of the *Enterobacteriaceae* family. This dysbiotic state compromises the integrity of the intestinal epithelial barrier, facilitating the translocation of lipopoly-saccharides (LPS) and other immunogenic molecules into systemic circulation. The ensuing chronic inflammatory environment is believed to influence neural functioning, contributing to both affective disturbances and cognitive decline [40].

Future research should move beyond descriptive models and aim to integrate microbiota-related findings with neurocognitive profiles and behavioral markers. Qualitative assessments that consider disease phase, patient insight, treatment adherence, and mood disturbances will be essential for constructing more comprehensive, explanatory models of gut–brain interactions in CD.

### 2.6 Neurocognitive Disorders in Crohn’s Disease Persons

Neurocognitive disorders are defined by impairments in memory, attention, executive functioning, and processing speed, often accompanied by emotional dysregulation. These deficits can markedly interfere with daily functioning and diminish overall quality of life. In individuals with inflammatory bowel disease (IBD), neuropsychological disturbances—including cognitive dysfunction—are increa-singly recognized [41]. Emerging neuroimaging evidence points to structural and functional brain alterations that may underlie these impairments. For instance, reductions in gray matter volume have been reported in key regions responsible for information processing and memory, such as the cingulate cortex and parahippocampal cortex, alongside widespread white matter hyperintensities [42].

Functional imaging studies further support these findings, revealing altered global brain network organi-zation and disrupted regional connectivity in patients with IBD [43]. These disruptions not only correlate with cognitive deficits but also frequently co-occur with depression and anxiety, suggesting a complex interplay between neurobiological and psychological factors [41]. However, it remains essential to differentiate cognitive impairment directly attributable to IBD—whether driven by inflammation or neurodegenerative mechanisms—from deficits that may arise secondarily due to psychiatric comorbidities or reduced insight into the disease. Poor insight, often linked with depressive symptoms, may contribute to suboptimal adherence to treatment regimens (*e.g*., dietary restrictions, medication compliance), potentially exacerbating disease progression and further impairing cognitive functioning [44]. Therefore, inter-preting cognitive symptoms in IBD should move beyond mere quantification to incorporate a critical, integrative approach—one that considers disease stage, emotional state, neurocognitive integrity, and treatment adherence—to develop more meaningful and patient-centered models of dysfunction [45].

### 2.7 Neuropsychological Assessment and Cognitive Impairments in IBD patients

Neuropsychological assessment plays a crucial role in the comprehensive evaluation of patients with Inflammatory Bowel Disease (IBD), offering a structured means to detect cognitive impairment across multiple domains. Commonly employed tools include:

• Montreal Cognitive Assessment (MoCA): A global cognitive screening instrument, particularly sensitive in detecting mild cognitive impairment (MCI) [46].

• Trail Making Test (TMT-A and TMT-B): Assesses processing speed, selective attention, and cognitive flexibility [47].

• Digit Span (Forward and Backward): Measures working memory and attentional capacity [48, 49].

• Repeatable Battery for the Assessment of Neuropsychological Status (RBANS): Evaluates learning, memory, visuospatial skills, and language [50].

• Stroop Color-Word Test (SCWT): Assesses inhibitory control, selective attention, and mental flexibility [46].

These assessments should be complemented by validated self-report measures of anxiety, depression, and perceived stress, which are highly prevalent in IBD and significantly influence cognitive functioning [51]. It is important to go beyond mere quantification of deficits, seeking instead to contextualize and qualify cognitive complaints according to disease phase, psychological comorbidities, and patient insight.

Emerging evidence suggests that chronic inflammation in IBD may adversely affect the central nervous system *via* the gut–brain axis, promoting neuroinflammation, altering gut microbiota composition, and impairing blood–brain barrier integrity [52]. The recognition of neuroinflam-matory biomarkers in IBD further supports the systemic nature of the disease and its capacity to affect cognitive and emotional regulation [53].

Importantly, cognitive impairment in IBD should not be viewed exclusively as a secondary effect of chronic illness or emotional distress. Increasing data points to the possibility that neuroinflammatory processes and gut dysbiosis may serve as primary contributors to cognitive dysfunction [54]. This distinction is essential, as it shifts the focus toward biological underpinnings rather than attributing symptoms solely to psychosocial factors.

Moreover, cognitive deficits and impaired insight into illness may mutually reinforce one another. Poor insight can hinder treatment adherence (*e.g*., dietary compliance, medication use), potentially exacerbating disease progression and contributing to further cognitive decline. Conversely, mood disorders such as depression—commonly seen in IBD—can both stem from and contribute towards neurocognitive dysfunction, thereby forming a complex, bidirectional relationship.

To address these multidimensional challenges, a multidisciplinary approach is warranted. This includes not only standardized neuropsychological assessment, but also proactive inflammation management, modulation of the gut microbiota, and psychological support aimed at improving insight, adherence, and emotional well-being. Such integrative care may be essential to preserving cognitive function and enhancing long-term quality of life in IBD patients. [55].

The main neuropsychological tools used in IBD research and clinical practice are summarized in Table **1**.

### 2.8 Critical Appraisal of the Current Literature

Despite the growing number of studies linking Crohn’s disease to neuropsychological dysfunction, several methodological limitations must be acknowledged. Many studies rely on cross-sectional designs, limiting the ability to draw causal inferences regarding the relationship between inflammation, mood disturbances, and cognitive impairment. Furthermore, sample sizes are often small, heterogeneous, and lack stratification by disease phase or psychological engagement level.

Additionally, while several psychometric tools have been validated in IBD populations, there remains a lack of consensus on how best to differentiate between primary neuroinflammatory cognitive deficits and those secondary to psychological distress or impaired insight. Many studies do not control for confounding variables such as medication use, nutritional status (*e.g*., B12 deficiency), or comorbid psychiatric conditions. The overlap between cognitive symptoms and mood disorders—particularly depression—also complicates interpretation, as few studies incorporate both neurocognitive testing and structured psychiatric interviews.

These limitations highlight the need for longitudinal, multimodal studies that integrate neuropsychological testing, inflammatory biomarkers, imaging, and behavioral assessments, ideally across different stages of disease progression and therapeutic engagement.

### 2.9 Anxiety and Depression in Crohn’s Disease: The Psychological Impact

The increasing global prevalence of Crohn’s disease (CD) necessitates a comprehensive and integrative approach that extends beyond its physiological under-pinnings to include its cognitive and psychological dimen-sions. Among the most prevalent comorbidities, anxiety and depression have been widely documented, exerting a profound influence on patients' disease perception, insight, treatment adherence, and overall quality of life [28]. These affective symptoms often manifest early, sometimes even at diagnosis, and persist throughout the clinical course, frequently in association with chronic fatigue, persistent pain, and a perceived loss of control.

Recent large-scale comparative studies have empha-sized how the burden of chronic inflammatory conditions like CD can exacerbate psychological vulnerability, increasing the risk for both systemic complications and neurocognitive dysfunction [53]. In this context, the gut–brain axis has emerged as a pivotal framework for understanding how psychological stress can both result from and contribute towards immune dysregulation and intestinal inflammation in inflammatory bowel disease (IBD). Psychological distress is thus increasingly recognized not merely as a consequence of chronic illness, but as an active modulator of disease progression, capable of influencing both inflammatory pathways and cognitive functioning.

Psychoneuroimmunological research has further revealed that dysregulations in the hypothalamic– pituitary–adrenal (HPA) axis and neuro-immune inter-actions may affect not only mood but also cognitive performance, potentially impairing insight and self-awareness regarding the disease [52]. These impairments can, in turn, reduce treatment adherence, creating a bidirectional cycle between poor disease control and neuropsychological deterioration. It becomes crucial, therefore, to differentiate between cognitive deficits primarily stemming from organic inflammatory or neuro-degenerative factors and those secondarily resulting from depression, psychological maladjustment, or reduced insight into the illness.

This distinction has significant clinical implications: cognitive complaints should not merely be quantified but qualified and contextualized within the patient’s stage of disease and level of psychological engagement. For instance, lower insight and executive dysfunctions are often reported even in patients with stabilized disease activity, suggesting that neurocognitive factors may operate independently of inflammatory markers and reflect a persistent vulnerability.

In response to these complexities, early psychological screening and personalized mental health support should be systematically integrated into IBD management pathways. One particularly effective conceptual framework is the Patient Health Engagement (PHE) Model developed by Graffigna and colleagues [56]. This model outlines a four-phase psychological trajectory—Blackout, Arousal, Adhesion, and Eudaimonic Project—each representing a distinct pattern of emotional response, cognitive pro-cessing, and behavioral orientation toward the illness. Importantly, these phases also reflect differing degrees of disease insight and readiness to engage with therapeutic regimens.

Clinicians who accurately assess a patient’s position within the PHE continuum can better tailor their communi-cation strategies, therapeutic goals, and psychological interventions. This not only promotes greater treatment adherence but also supports cognitive resilience and enhances the patient’s capacity for meaningful disease integration and improved quality of life.

### 2.10 Psychological Assessment in InflammatoryBowel Disease (IBD): Emphasis on Anxiety, Depression, and Alexithymia

Growing awareness of the intricate interplay between psychological and physiological factors in inflammatory bowel disease (IBD) has prompted greater emphasis on integrating psychological evaluation into standard clinical care. IBD, encompassing both Crohn’s disease (CD) and ulcerative colitis (UC), is frequently accompanied by psychological comorbidities such as anxiety, depression, and alexithymia—defined as difficulty in identifying and expressing emotions [54]. These conditions not only impair quality of life but may also influence disease activity through both behavioral and neurobiological pathways, including altered stress responses and immune modulation [55, 56].

Furthermore, psychological distress can distort symptom perception, reduce insight into the disease, and diminish adherence to treatment regimens, including dietary restrictions and pharmacological therapies [57, 58]. This reduced adherence, in turn, may hinder disease stabilization and contribute to further disease progression and cognitive impairment, particularly in domains such as memory, executive function, and attention [59, 60].

Given the bidirectional nature of the gut–brain axis, and the emerging evidence that neurocognitive dysfunction may both reflect and exacerbate mood disturbances in IBD [61, 62], the incorporation of validated and stage-sensitive psychological and cognitive assessment tools is crucial. Such tools can help distinguish between primary cognitive deficits—arising from inflammatory or neurodegenerative processes—and secondary deficits related to low mood or reduced insight into illness. Ultimately, a qualitative as well as quantitative approach to symptom assessment may allow for the development of integrated care models tailored to the phase and severity of disease progression.

### 2.11 Psychometric Tools in Clinical Use

A variety of standardized psychometric instruments are employed to assess psychological functioning in patients with Inflammatory Bowel Disease (IBD), reflecting the complex interplay between mood disturbances, cognitive impairments, and disease insight. An overview of the most widely used psychological assessment tools in this population is presented in Table **2**. Among these, the **Hospital Anxiety and Depression Scale (HADS)** [53] and the **Beck Depression Inventory (BDI)** [54] are frequently utilized to evaluate symptoms of anxiety and depression. For rapid screening of generalized anxiety, the **Generalized Anxiety Disorder-7 (GAD-7)** [55] offers a concise and effective measure.

To assess emotional awareness and regulation—processes closely tied to both mood and cognitive insight—the ** Toronto Alexithymia Scale (TAS-20)** [56] remains the gold standard. In one study [57], the combined use of HADS and TAS-20 revealed a robust correlation between alexithymia and depressive symptoms. This association has been interpreted as evidence of disrupted interoceptive processing and impaired affect regulation, which in turn may contribute to the misinterpretation of somatic signals. These mechanisms can intensify both physical discomfort and emotional distress, potentially interfering with treatment adherence and reinforcing cycles of poor disease insight.

While these tools provide valuable quantitative data, a more integrated, phase-specific interpretation of the psychological profiles they reveal is needed. Especially in the context of IBD, where neuroinflammation and mood disorders may converge, it is crucial to discern whether observed cognitive and affective alterations stem from primary neurological processes or are secondary to psychological factors such as poor insight or behavioral disengagement from treatment.

### 2.12 Clinical Recommendations for Psychological Screening and Integration in IBD Care

Based on the evidence reviewed, we recommend the routine integration of psychological screening and support into standard clinical pathways for Crohn’s disease, specifically:

#### • When to Screen

Psychological evaluation should be conducted at diagnosis, during major disease transitions (*e.g*., relapse, treatment changes), and at regular intervals, even in clinical remission.

#### • Who Should Screen

A trained clinical psychologist or neuropsychologist should administer and interpret validated instruments, ideally as part of a multidisciplinary IBD team.

#### • Recommended Tools

Initial screening: HADS, GAD-7, TAS-20, PHE Scale. Cognitive screening: MoCA, TMT-A/B. In-depth assessment: RBANS, Stroop Test, DSQ, structured clinical interviews (*e.g*., SCID-5).

#### • Clinical Integration

Psychological findings should be regularly discussed in multidisciplinary meetings to guide therapeutic adjust-ments. Referral pathways should be clearly defined to facilitate timely access to mental health care. Engagement strategies (*e.g*., based on the Patient Health Engagement model) should be tailored to the patient’s phase-specific profile of emotional reactivity, cognitive function, and insight into illness. These practices aim to identify at-risk patients early, personalize care, and promote adherence, resilience, and better clinical outcomes.

### 2.13 Emotional Dysregulation and Defense Mechanisms

Building on this, Reilly *et al*. investigated how immature defense mechanisms and elevated alexithymic traits may hinder the ability to differentiate between emotional and somatic experiences. These psychological characteristics likely contribute to reduced insight into one's emotional state and a delay in seeking psychological support. Their findings underscore the importance of integrating emotion-focused strategies into standard IBD care, as emotional dysregulation may compromise not only psychological adjustment but also treatment adherence [56].

Similarly, Viganò *et al*. reported significant associations between alexithymia, psychiatric symptomatology, and illness perceptions, employing validated instruments such as the Beck Depression Inventory (BDI) and the Toronto Alexithymia Scale (TAS-20) [57]. Their results highlight how emotional dysregulation and limited insight into emotional states increase psychological vulnerability and foster maladaptive illness representations. These factors may further contribute to impaired self-awareness and poor disease insight, ultimately affecting coping strategies and adherence to treatment protocols [56]. These findings support a broader interpretative model in which neuro-cognitive dysfunction, mood disturbance, and impaired insight interact dynamically across different phases of the disease [57].

### 2.14 Toward a Tailored Psychological Assessment Model

Further research underscores the relevance of assessing cognitive–affective coping styles and unconscious defense mechanisms in individuals with IBD, as these factors may contribute to neurocognitive dysfunction and diminished insight into the disease. The proposed model supports the need for tailored psychological assessments that go beyond symptom quantification to capture qualitative aspects of patient experience, such as maladaptive coping strategies and alexithymic traits, which have been associated with increased disease burden [59, 60]. Moreover, these psychological variables may influence treatment adherence and exacerbate disease progression through their impact on mood and cognition [63, 64]. Incorporating tools like the Defense Style Questionnaire (DSQ) [60] can help identify these underlying psychological patterns, providing valuable insights into the interplay between mood disturbances, cognitive performance, and insight. This, in turn, can support more accurate clinical decision-making and facilitate the development of individualized therapeutic plans based on the phase or stage of the disease [65].

### 2.15 The Psychoneuroimmunological Perspective

Introducing a neurobiological dimension, Vinni *et al*. [59] investigated the association between TAS-20 scores and inflammatory biomarkers, such as C-reactive protein (CRP) and proinflammatory cytokines. Their findings support the hypothesis that alexithymia may represent not only a psychological vulnerability but also a psycho-neuroimmunological mechanism contributing to systemic inflammation. This aligns with the broader concept that emotional processing deficits—often observed in individuals with impaired insight—may play a role in both psychological distress and neurocognitive dysfunction [14, 37, 44]. Such deficits could result from inflammatory mechanisms or, conversely, emerge secondarily due to poor insight and low treatment adherence, particularly in patients experiencing mood disturbances [25, 41, 60]. These interactions may contribute to disease progression in IBD, reinforcing the need to differentiate between primary (biological) and secondary (psychological or behavioral) origins of cognitive impairment [29, 47]. Therefore, understanding the relationship between emotional dysregulation, insight, and immune responses is essential for interpreting cognitive symptoms within a disease-stage-dependent framework. This supports the relevance of integrative biopsychosocial models in advancing both clinical understanding and individualized treatment strategies in IBD [12, 36, 59].

### 2.16 Clinical Implications and Integrated Assessment Strategies

Taken together, the findings underscore the necessity of a comprehensive and interdisciplinary approach to psychological assessment in inflammatory bowel disease (IBD). The use of validated instruments for detecting anxiety, depression, and alexithymia—alongside clinical parameters and biological markers—not only enhances risk stratification but also facilitates the development of personalized, phase-specific interventions. These tools should be interpreted in conjunction with cognitive functioning and insight into illness, as these domains are often intertwined and may differentially influence treatment adherence, particularly in the presence of depressive symptoms and neurocognitive impairments.

Importantly, future approaches should distinguish cognitive deficits arising from inflammatory and neuro-biological mechanisms from those that are secondary to psychological factors such as depression or low insight into illness. These distinctions are critical, as they inform tailored interventions and may shed light on the bidirectional relationship between psychological distress and disease progression.

Furthermore, diminished adherence to dietary and pharmacological regimens may not only reflect behavioral resistance but also signal underlying neurocognitive dysfunction, which in turn may exacerbate disease activity and contribute to further cognitive decline—a process that remains underexplored and warrants longitudinal investigation. Indeed, cognitive deficits secondary to depression may impair insight into the disease, further hindering treatment adherence and quality of life.

While the authors highlight several potential models of neurodegeneration associated with IBD, a more critical synthesis of these mechanisms are required. Specifically, future research should examine the extent to which mood disturbances contribute towards cognitive dysfunction, and whether these changes persist independently of inflammatory activity or are reversible with psychological intervention.

Rather than focusing solely on the quantification of cognitive or emotional symptoms, a more nuanced, integrative framework is necessary—one that considers the temporal dynamics of the disease, the patient's psychological profile, and the stage-specific relevance of symptoms. This approach would support a more accurate interpretation of patient complaints and inform clinical decision-making.

In recent years, the contribution of psychological interventions to the integrated care of patients with IBD has gained increasing recognition within both clinical and research communities. Although IBD has traditionally been managed within a medical and gastroenterological framework, substantial evidence now underscores the role of psychological distress, emotional dysregulation, and the chronic disease burden as key factors that exacerbate symptom activity, reduce health-related quality of life, and compromise adherence to therapeutic regimens.

As a result, contemporary multidisciplinary care models are increasingly incorporating psychological assessment and intervention as core components of treatment protocols. This shift is consistent with the biopsychosocial paradigm, which recognizes the complex interplay of biological, psychological, and social factors in chronic disease and highlights the indispensable role of clinical psychologists in integrated care teams.

This integrative model holds promise for reducing psychological distress, modulating inflammatory responses, improving insight and treatment adherence, and ultimately enhancing both emotional well-being and clinical outcomes in the management of IBD [61].

### 2.17 Cognitive Behavioral Therapy (CBT)

Among the psychological interventions utilized in the management of inflammatory bowel disease (IBD), cognitive behavioral therapy (CBT) remains the most extensively validated and empirically supported approach [62]. CBT targets maladaptive cognitive patterns, emotional dys-regulation, and behavioral responses that may exacerbate disease burden or hinder effective disease management. Notably, recent evidence has shown that CBT not only alleviates symptoms of anxiety and depression [60], which are commonly comorbid with IBD, but also contributes to improved treatment adherence, enhanced disease-related insight, and more effective self-management behaviors [64].

For instance, Hunt *et al*. [62] reported that structured CBT programs significantly improved emotional functioning, strengthened coping mechanisms, and facilitated more proactive engagement with medical care. These findings suggest that CBT may mitigate the cognitive and affective consequences of chronic inflammation and disease-related distress, potentially reducing the risk of cognitive impairments associated with low insight or poor adherence.

Moreover, CBT’s adaptability across diverse patient populations—including pediatric, adolescent, and adult cohorts—as well as its delivery *via* digital platforms, reinforces its relevance as an integrative tool in IBD care. Importantly, given the dynamic course of IBD, CBT may also support patients differently depending on the phase or stage of the disease, offering tailored strategies to address evolving psychological and neurocognitive needs.

### 2.18 Mindfulness-based Interventions

Mindfulness-based therapies are increasingly recognized as evidence-based interventions for individuals with inflammatory bowel disease (IBD), particularly due to their capacity to address both psychological distress and cognitive-emotional dysregulation. By fostering a non-judgmental awareness of thoughts, emotions, and bodily sensations, these therapies may enhance patients’ ability to manage stress and physical discomfort, which are often associated with disease flare-ups and reduced quality of life. Importantly, such interventions could also influence insight into the illness and improve adherence to treatment regimens by promoting greater self-awareness and emotional resilience [ 60 ]. A recent evaluation of a blended online mindfulness program for individuals with IBD reported significant improvements in emotional regulation, perceived stress, and overall patient satisfaction. Participants rated the intervention as both accessible and acceptable, underscoring its feasibility for broader clinical application—particularly within digitally delivered, integrative care frameworks. These findings suggest that mindfulness-based approaches may not only alleviate affective symptoms but also contribute to maintaining cognitive integrity and treatment engagement, especially in patients at risk of reduced insight and poor disease management [60].

### 2.19 Acceptance and Commitment Therapy (ACT)

Acceptance and Commitment Therapy (ACT) has emerged as a promising psychological intervention for individuals experiencing distress associated with chronic illnesses, such as Crohn’s disease (CD) and other forms of inflammatory bowel disease (IBD). ACT integrates mindfulness and acceptance-based strategies to enhance psychological flexibility—defined as the capacity to pursue valued life goals even in the presence of pain, discomfort, or difficult emotions. This therapeutic flexibility is particularly relevant in the context of chronic diseases, where patients often face ongoing physical symptoms and psychological burdens that can negatively affect mood, quality of life, and cognitive functioning.

Carvalho *et al*., in a randomized controlled trial, demonstrated that ACT significantly reduced psychological distress and improved adaptive functioning in patients with IBD. Notably, ACT may be especially beneficial for patients exhibiting high levels of experiential avoidance or persistent health-related anxieties [64], both of which have been associated with impaired insight and poor adherence to treatment.

Moreover, given the complex interplay between mood disturbances, cognitive deficits, and disease-related insight, ACT may contribute indirectly to cognitive functioning by targeting depressive symptoms and improving engagement with treatment protocols. As cognitive deficits in CD may arise from inflammatory, neuropsychological, or insight-related mechanisms, therapeutic approaches like ACT—when integrated into a broader psychosocial care model—can help contextualize patient complaints not just quantitatively, but qualitatively, in line with the phase or severity of the disease.

### 2.20 Psychosomatic Psychotherapy and the Biopsychosocial Perspective

There has been a renewed interest in psychosomatic psychotherapy, which aligns closely with the biopsycho-social model of illness and emphasizes the inter-connectedness of emotional, cognitive, and physiological processes. This therapeutic approach explores the interplay between unresolved emotional conflict, the symbolic expression of bodily symptoms, and the patient’s coping strategies, which may include limited insight into the disease and its management [1, 2, 3]. A pilot study conducted by D’Onofrio *et al*. [65], published in *Minerva Gastroenterology*, examined patients undergoing biological therapies and found that incorporating a structured psychosomatic assessment allowed for a more nuanced identification of emotional distress, relational difficulties, and psychosomatic dynamics that may influence disease progression [4, 5]. Importantly, this approach also aids in differentiating cognitive deficits potentially arising from inflammatory processes from those rooted in psychological factors or low disease insight [2, 3]. Psychosomatic psychotherapy places strong emphasis on the therapeutic alliance, the development of emotional insight, and the symbolic interpretation of somatic symptoms, all of which are crucial for improving adherence to treatment and enhancing patient outcomes [6, 7]. As such, it may be particularly relevant for patients with chronic inflammatory diseases who often experience cognitive dysfunction, mood disturbances, and a sense of alienation within a strictly biomedical model of care [1, 4, 7].

### 2.21 Comparative Overview of Psychological Interventions in Crohn’s Disease

This conceptual framework illustrates the inter-connections among gut dysbiosis, chronic inflammation, neurocognitive dysfunction, mood disturbances, and disease insight. It also highlights the therapeutic targets where psychological interventions—such as CBT, ACT, mindfulness, and psychosomatic psychotherapy—may act to improve adherence and overall outcomes.

While various psychological interventions have demonstrated benefit in Crohn’s disease, selecting the most appropriate approach requires a phase-specific and patient-centered strategy. CBT remains the most empirically validated option, particularly effective in treating mood disorders and improving adherence. ACT, although less studied in IBD, shows promise in addressing experiential avoidance and chronic emotional burden. Mindfulness-based programs are particularly suited to patients with high emotional reactivity or difficulty tolerating physical symptoms. Finally, psychosomatic psychotherapy offers a more interpretative and relational model, especially for patients with limited insight, somatization, or unelaborated emotional trauma.

Integrating these approaches within multidisciplinary care teams and matching them to specific psychological profiles may enhance engagement, reduce dropout rates, and optimize therapeutic outcomes.

### 2.22 Final Considerations and Clinical Integration

Overall, current evidence supports the effectiveness of psychological therapies—particularly cognitive behavioral therapy (CBT), acceptance and commitment therapy (ACT), mindfulness-based interventions, and psycho-somatic approaches—in enhancing both emotional well-being and clinical outcomes in patients with inflammatory bowel disease (IBD) [1]. These interventions address prevalent comorbidities such as depression, anxiety, and stress, while also fostering resilience, emotional self-regulation, and the capacity to cope with the uncertainty and lifestyle disruptions inherent to chronic illness [5]. However, to fully understand their impact, it is essential to consider the interaction between mood alterations and cognitive functioning, especially as cognitive deficits and impaired insight may significantly influence treatment adherence and disease progression [3, 4]. In particular, distinguishing cognitive deficits arising from inflammatory or biological mechanisms from those associated with psychological processes or reduced illness insight is crucial [5]. Moreover, poor adherence to IBD treatments—including dietary, behavioral, and pharmaco-logical components—may further compromise disease stabilization and exacerbate cognitive impairments detectable in neuropsychological assessments [5]. These cognitive deficits, often secondary to mood disorders such as depression, may in turn reinforce poor insight and reduce motivation for self-care [5]. Although the current literature proposes several explanatory models for neurodegeneration in IBD, many lack critical evaluation, often offering descriptive overviews without integrating these models into a coherent framework for clinical interpretation [5]. Importantly, mood-related changes should be analyzed not only in terms of their prevalence but also in their potential impact on cognitive performance and disease management [6]. Thus, beyond the quantification of psychological symptoms, it is vital to contextualize and interpret patient-reported complaints within a dynamic model that accounts for the disease phase and its neuropsychological and behavioral consequences [7]. Integrating these therapeutic modalities into routine care—ideally through interdisciplinary teams—facilitates a holistic, person-centered approach that aligns more accurately with the complex, multifactorial nature of IBD [1, 7].

### 2.23 Integrated Model of Psychoneuroimmunological Dysfunction in Crohn’s Disease

To synthesize the multifaceted findings discussed throughout this review, we propose a conceptual framework that integrates biological, psychological, and behavioral domains in Crohn’s disease. This model illustrates the bidirectional relationships among systemic inflammation, gut microbiota imbalance, neurocognitive dysfunction, mood disturbances, and disease insight. It also highlights the role of psychological interventions as potential modulators of these interactions.

### 2.24 Future Research Directions

While substantial progress has been made in elucidating the psychological and neurocognitive dimensions of Crohn’s disease, several critical research gaps remain. We propose the following future research priorities:

**1**. **Longitudinal studies** evaluating whether neurocognitive impairments precede, follow, or co-evolve with changes in inflammatory activity and mood disorders across different disease stages.

**2**. **Randomized controlled trials (RCTs)** comparing the efficacy of CBT, ACT, mindfulness-based interventions, and psychosomatic psychotherapy in improving insight, cognitive function, and treatment adherence.

**3**. **Multimodal studies** integrating neuroimaging, neuropsychological testing, and microbiome profiling to identify biomarkers of cognitive vulnerability or resilience in CD patients.

**4**. **Phase-specific intervention models**, tailored to levels of insight, mood stability, and cognitive integrity—*e.g*., testing whether early psychological engagement alters disease trajectory or neurocognitive decline.

**5**. **Digital therapeutics trials** assessing the feasibility and clinical efficacy of remotely delivered, personalized psychological interventions (*e.g*., AI-based CBT coaching, mobile mindfulness programs) in maintaining cognitive-emotional stability and adherence.

**6**. **Neuropsychological rehabilitation protocols** specifically designed for CD patients with persistent executive or memory deficits, aiming to restore cognitive function and improve disease self-management.

These lines of inquiry are essential to move beyond descriptive associations and toward actionable, personalized care models for Crohn’s disease that integrate biological, psychological, and cognitive dimensions.

## CONCLUSION

The Gut-Brain Axis in Crohn’s Disease: From Understanding to Intervention

Crohn’s disease (CD), once considered a localized gastrointestinal disorder, is now understood as a complex systemic condition with significant psychological, neuro-cognitive, and emotional dimensions. Accumulating evidence indicates that patients with CD are at increased risk for anxiety, depression, cognitive dysfunction, and alexithymia compared to the general population [1, 2, 3].

Disruptions in the gut-brain axis—through mechanisms involving neuroinflammation, immune activation, and microbiota dysbiosis—appear to contribute to these impair-ments [5]. Neuroimaging and cognitive assessments have revealed functional and structural brain changes in CD patients [6], yet the interpretation of these findings often lacks integration with the patient’s psychological profile and level of disease insight.

It is increasingly evident that mood disturbances in CD are not only comorbidities but may actively influence cognitive performance and disease insight [7]. Depression, for example, is frequently associated with impaired executive functioning and poor treatment adherence [8]. Insight into the disease, in turn, may be partially dependent on preserved neurocognitive function, suggesting a complex, bidirectional relationship between mood, cognition, and treatment behavior [9].

This highlights the importance of differentiating between primary neurocognitive impairments—potentially linked to inflammatory or neurobiological processes—and secondary deficits arising from psychological distress or reduced disease awareness [10]. Poor adherence to dietary and pharmacological interventions may also exacerbate disease progression, creating a feedback loop that reinforces cognitive and emotional decline [11].

Validated clinical tools such as the Hospital Anxiety and Depression Scale (HADS), Toronto Alexithymia Scale (TAS-20), and Montreal Cognitive Assessment (MoCA) are effective for early identification of psychological and cognitive challenges in CD [12, 13]. Moreover, psycho-logical interventions—including Cognitive Behavioral Therapy (CBT), Acceptance and Commitment Therapy (ACT), and mindfulness-based strategies—have demonstrated promising outcomes in improving emotional regulation, insight, and engagement with treatment [14, 15].

However, despite encouraging progress, a critical gap remains in the standardization of assessment protocols and in the longitudinal evaluation of these interventions’ effects on disease trajectory. Future research should not only quantify impairments but also qualify and contextualize them within a broader model that considers disease phase, patient insight, and behavioral factors [16].

Integrating psychological assessment and care into routine gastroenterological practice—*via* a biopsychosocial and multidisciplinary model—may improve patient outcomes, reduce cognitive and emotional burden, and enhance overall adherence and quality of life [17].

## Figures and Tables

**Table 1:** Neuropsychological assessment tools in Crohn’s disease and IBD patients.

**Test Name**	**Cognitive Function Assessed**	**Context of Use in IBD/CD**
Montreal Cognitive Assessment (MoCA)	Global screening for Mild Cognitive Impairment (MCI)	Early diagnosis of mild cognitive deficits is used in IBD patients
Trail Making Test (TMT-A and TMT-B)	Selective attention, cognitive flexibility	Assesses processing speed and cognitive switching, which are often affected in Crohn’s disease
Digit Span (Forward and Backward)	Working memory and attention	Used to analyze verbal memory functions in IBD patients
Repeatable Battery for the Assessment of Neuropsychological Status (RBANS)	Memory, language, learning	Multidomain test useful for evaluating various cognitive areas in patients with Crohn’s disease
Stroop Color and Word Test (SCWT)	Cognitive inhibition, processing speed	Useful for evaluating executive function deficits and emotional interference

**Table 2:** Psychological assessment in inflammatory bowel disease (IBD): Emphasis on anxiety, depression, and alexithymia.

**Test Name**	**Function**	**Context of Use in IBD/CD**
Hospital Anxiety and Depression Scale (HADS)	Assesses symptoms of anxiety and depression	Used to monitor emotional comorbidities in Crohn's and UC patients
Beck Depression Inventory (BDI)	Measures the severity of depressive symptoms	Applied to assess depression in relation to quality of life in IBD patients
Generalized Anxiety Disorder-7 (GAD-7)	Rapid screening for generalized anxiety	Used as a brief tool to detect anxiety states in patients
Toronto Alexithymia Scale (TAS-20)	Assesses difficulty in identifying and expressing emotions	Particularly helpful for identifying alexithymia, which is common in IBD patients
Defense Style Questionnaire (DSQ)	Analyzes psychological defense mechanisms	Useful in detecting dysfunctional defense styles related to somatization and coping in IBD
Patient Health Engagement Scale (PHE)	Measures psychological engagement and resilience	Assesses the patient’s adaptation stage to chronic illness

**Table 3:** Comparative overview of psychological interventions in Crohn’s disease.

** Intervention **	** Strengths **	** Limitations **	** Best Indications **
** Cognitive Behavioral Therapy (CBT) **	Well-studied; improves mood, adherence, and coping	Requires cognitive engagement and structured sessions	Patients with anxiety, depression, and adequate insight
** Acceptance and Commitment Therapy (ACT) **	Improves psychological flexibility, acceptance	Less evidence in IBD-specific settings	Patients with avoidance behaviors, chronic distress
** Mindfulness-Based Interventions **	Effective in stress reduction and emotion regulation	Variable adherence; best with motivated patients	Patients with high reactivity or somatic amplification
** Psychosomatic Psychotherapy **	Explores symbolic meanings and emotional conflicts	Requires longer duration; less standardized	Patients with alexithymia, insight deficits, or resistance to CBT models

**Table 4:** Psychological model.

**Approach**	**Core Features**	**Clinical Application in IBD/CD**
Cognitive Behavioral Therapy (CBT)	Identifies and restructures maladaptive thoughts and behaviors	Improves emotional well-being, coping skills, and treatment adherence in patients with CD
Mindfulness-Based Interventions	Promotes non-judgmental awareness of present-moment experiences	Reduces stress and emotional reactivity; accessible in both face-to-face and online formats
Acceptance and Commitment Therapy (ACT)	Combines mindfulness with value-based action and acceptance strategies	Enhances psychological flexibility; effective in managing chronic illness-related distress
Psychosomatic Psychotherapy	Explores the emotional-symbolic meaning of physical symptoms and illness experience	Useful in long-standing IBD; integrates emotional insight and therapeutic alliance

## LIST OF ABBREVIATIONS

ACT: Acceptance and Commitment Therapy
AIEC: Adherent-Invasive Escherichia Coli
BDI: Beck Depression Inventory
CD: Crohn’s disease
CNS: central nervous system
CRP: C-reactive protein
DSQ: Defense Style Questionnaire
GAD-7: Generalized Anxiety Disorder-7
HADS: Hospital Anxiety and Depression Scale
HPA: hypothalamic–pituitary–adrenal
IBD: inflammatory bowel diseases
IBDU: inflammatory bowel disease unclassified
IC: indeterminate colitis
LPS: lipopolysaccharides
MAP: Mycobacterium avium subspecies paratuberculosis
MoCA: Montreal Cognitive Assessment
PHE: Patient Health Engagement
TAS-20: Toronto Alexithymia Scale
TNF-α: anti–tumor necrosis factor-alpha
UC: ulcerative colitis
